# Maxillary and mandibular dental arch forms in a Jordanian population with normal occlusion

**DOI:** 10.1186/s12903-021-01461-y

**Published:** 2021-03-09

**Authors:** M. Aljayousi, S. Al-Khateeb, S. Badran, E. S. Abu Alhaija

**Affiliations:** 1 Division of Orthodontics, Department of Preventive Dentistry, Faculty of Dentistry, Jordan University of Science and Technology, P.O. Box 3030, Irbid, 22110 Jordan; 2 Department of Orthodontics, Faculty of Dentistry, The University of Jordan, Amman, Jordan; 3 College of Dental Medicine, Qatar University, P.O.Box 2713, Doha, Qatar

## Abstract

**Background:**

Ethnic background has been claimed to affect arch form. The purpose of this study was to describe and elucidate the maxillary and mandibular arch forms in Jordanian population and to develop a classification method for these forms which could be employed to construct orthodontic archwires accordingly.

**Methods:**

The sample was comprised of study casts of five hundred and twenty subjects (231 males and 289 females with a mean age of 15.4 ± 1.02 years). All subjects had permanent dentition with normal occlusion. A mathematical method associated with a polynomial function of 6th degree was employed to assess the dental arch forms. The resultant arch forms were classified into 5 groups for both the maxilla and mandible utilizing a computer software with special code designed for this study. Each group was subdivided into 3 subgroup sizes namely: small, medium, and large.

**Results:**

Catenary arch form was found in 47% and 41.2% (*p* ˂ 0.01) of maxilla and mandible arch forms, respectively. Form 2 (which is halfway between ellipse and U-shaped arch form) was found in 27.7% and 26.7%. Medium size arch form was found in 55.4% of the maxillary and 65.6% of the mandibular arch forms.

**Conclusion:**

Catenary arch form was the most prevalent arch form, followed by wide elliptical form. The other forms, which included tudor arch, tapered equilateral and quadrangular forms were less frequent. Regarding size, the medium size was the most prevalent among the studied samples.

## Background

Several attempts have been made to define an “ideal” arch form taking into cognizance the fact that dental arch is symmetric in nature and can be represented by an algebraic or geometric formula [1].

The ideal lower arch form has been described as a slightly modified equilateral triangle with the base representing the intercondylar width; the six anterior teeth are arranged on the arc of a circle, and the radius is equivalent to the width of canines and incisors combined [2, 3]. Other suggested arch forms include semi-ellipse [4], parabolic [5], and catenary form [6–8]. Other authors have suggested different forms for upper and lower arches; with the upper arch taking the form of an ellipse, and the lower arch a parabola [9].

It has been suggested that there is presently no particular form that precisely describes dental arch forms, and customization of arch forms seems to be a requisite in many cases to achieve optimum long-term stability [1, 10].

Several factors have been claimed to affect arch size and forms such as ethnic background, type of malocclusion, variability in eruptive parts of the teeth, growth of the supporting bones, and movement of the teeth after emergence due to unwholesome habits and unbalanced muscular pressures [11–13]

Dental arch forms have been evaluated in different populations [14–17]. However, no studies have been conducted in Jordan. Accordingly, the aims of this study was to determine the maxillary and mandibular arch forms in Jordanian population and to report on the mean of dental arch dimensions for Jordanians.

## Methods

### Collection of data

A total of 6023 school students (2365 males and 3658 females), with an age range of 15–17 years, were examined in randomly selected schools from different districts in Jordan. The schools were randomly selected from a list obtained from the Directorate of Education in the north, center and south of the country. Four schools were selected from each city by selecting every third school in the list. Sample size calculation was done based on cross-sectional survey studies employing the sample size chart with the power of 0.90. The minimum number of subjects expected to be included in this study was 430.

A full clinical examination was conducted by one examiner in the school premises using a mouth mirror under natural lighting (MJ). Five hundred and twenty students (231 males and 289 females with mean age of 15.4 ± 1.02 years) fulfilled the following inclusion criteria and were invited to participate in the study; class I incisor and molar relationships, minimal crowding or spacing (≤ 2 mm), no or minor tooth rotations, no crossbite or scissors bite, all permanent teeth erupted except third molars, no missing or supernumerary teeth, no anomaly in size or shape of teeth, and no history of orthodontic treatment.

Upper and lower alginate impressions (Kromopan, Lascod s.p.a, Italy) and wax bite were taken. Impressions were kept according to the manufacturer’s recommendation and poured on the same day with hard dental stone (Zhermack Elite Ortho Stone, KAB Dental Inc., U.S.A) to produce orthodontic study models.

### Measurements

The dental casts were scanned employing HP Scanjet G4050 (Hewlett-Packard Company, Palo Alto, CA, USA) and images with 300 DPI resolution were obtained. The position of the dental casts on the scanner was established with a millimeter translucent paper specially designed for this purpose. It was accomplished by photocopying a sheet of millimeter paper on a transparent sheet.

The customized transparent sheet was placed between the scanner glass surface and the occlusal plane of the dental cast, so that the posterior edge of the dental cast would coincide with the abscissa axis (x) and the dental midline with the ordinate axis (y), thereby creating a Cartesian coordinate system.

For each cast image, 14 points were established on the dental arch representing the center of the clinical crown of the incisors, canines, buccal cusps of premolars, and the mesiobuccal cusps of the first and second molars. The perpendicular distance to the midline from each point was analyzed and resolved into an x and y component and measured in millimeters.

**Fig. 1:**
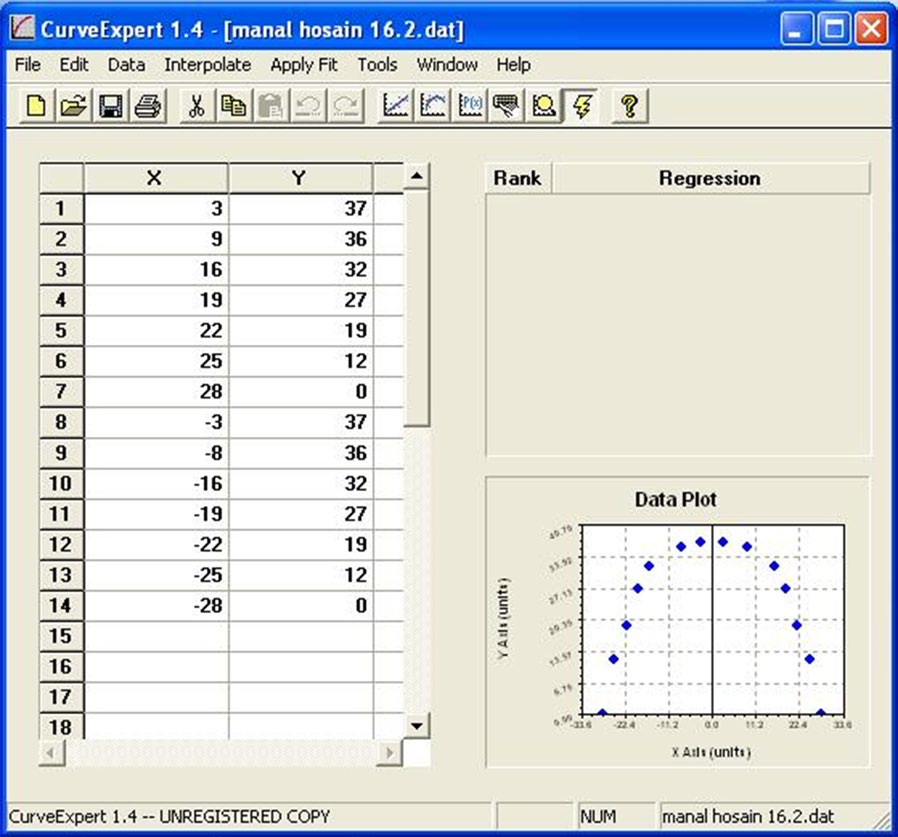
The function screen window of the x and y coordinates and a plot of one arch as displayed by the CurveExpert software

Measurements of the x and y coordinates of the 14 points of each dental cast image were plotted employing a computer software (CurveExpert, version 1.4, Hyams Development. U.S.A) in order to obtain the polynomial function that best describe the curve corresponding to the dental arch form (Fig. 1).

After scanning and analyzing all the dental casts, they were grouped into different arch forms according to the following procedures. Each photo was converted to a “DAT format” file, employing the CurveExpert software program. The photo then appeared as a set of points (indices x and y), which was established by the original photo. An interpolation was carried out on these sets of points using MATLAB (version 7.4.0.287 (R2007a), The MathWorks Inc., Natick, Massachusetts, U.S.A) to produce a polynomial function of the sixth order. Each set of points had 14 points. A curve containing 100 points was generated employing the function generated by interpolation. In order to achieve 100 steps on the x-axis, a specific type of scaling was carried out on each set of points. Thereafter, all curves were shifted to start from the same point, which was zero. The slope of each point relative to its neighbors was computed. The slope of each point in each curve was compared to the slope of the points which shared the same x values, x + 1 or x − 1 in all the other curves. The curves were then categorized into different groups based on the slopes of the points on each curve. The largest five groups (groups that had the most frequent types of curves) were christened the main groups.

Each of the maxillary and mandibular curves were also divided into three sizes within each arch form based on the transverse distance between the tips of the mesiobuccal cusps of the right and left second molars. The minimum distance was then subtracted from the maximum distance, and the difference was divided into three ranges. Each cast was classified into one of these three groups according to its width. The three ranges of distances between the second molars in each arch form of the maxilla and mandible are presented in Table 1.

**Table 1:** The range of inter-second-molar distances that determined arch size of each form

Arch size (mm)	Mandible			Maxilla		
	Small	Medium	Large	Small	Medium	Large
*Arch form*						
1	44–49	50–54	55–60	51–55	56–60	61–66
2	44–49	50–54	55–60	50–54	55–59	60–64
3	46–50	51–54	55–60	51–55	56–59	60–64
4	45–50	51–56	57–62	50–53	54–58	59–62
5	43–48	49–55	56–60	48–52	53–57	58–62

The intercanine width (from cusp tip to cusp tip), intermolar width (from the mesiobuccal cusp of the right first molar to the mesiobuccal cusp of the left first molar) and arch depth (the perpendicular line connecting the midpoint between the incisal edges and the transverse line passing through the distal surfaces of the first molars) were measured.

### Error of the method

Twenty (10 upper and 10 lower) randomly selected casts were reanalyzed and the arch parameters were remeasured after one month interval. Dahlberg’s formula for double determination was employed to calculate the standard error of the method [18]. Houston’s coefficient of reliability was also computed [19].

The error in measurement of the intercanine width, the intermolar width and the arch depth was 0.34 mm, 0.39 mm and 0.37 mm, respectively. Houston’s coefficient of reliability was above 92% for all the measured variables.

### Statistical analysis

Data analysis was carried out employing the Statistical Package for the Social Sciences (SPSS) software (SPSS 18.0, SPSS Inc., Chicago, USA). Mean and Standard deviations were computed for all the measured variables. Chi-square test was employed to investigate if there were differences between the frequency of the different arch forms and sizes in each of the maxilla and mandible. In order to compare the arch sizes in males and females, one-way univariate analysis of covariance (ANCOVA) was performed with gender as the fixed variable to detect any difference between the adjusted mean of the ages.

Analysis of variance (ANOVA) was employed to compare the arch parameters in the different arch forms. The p-value was predetermined at 0.05 as the level of significance.

## Results

### Arch forms

**Fig. 2:**
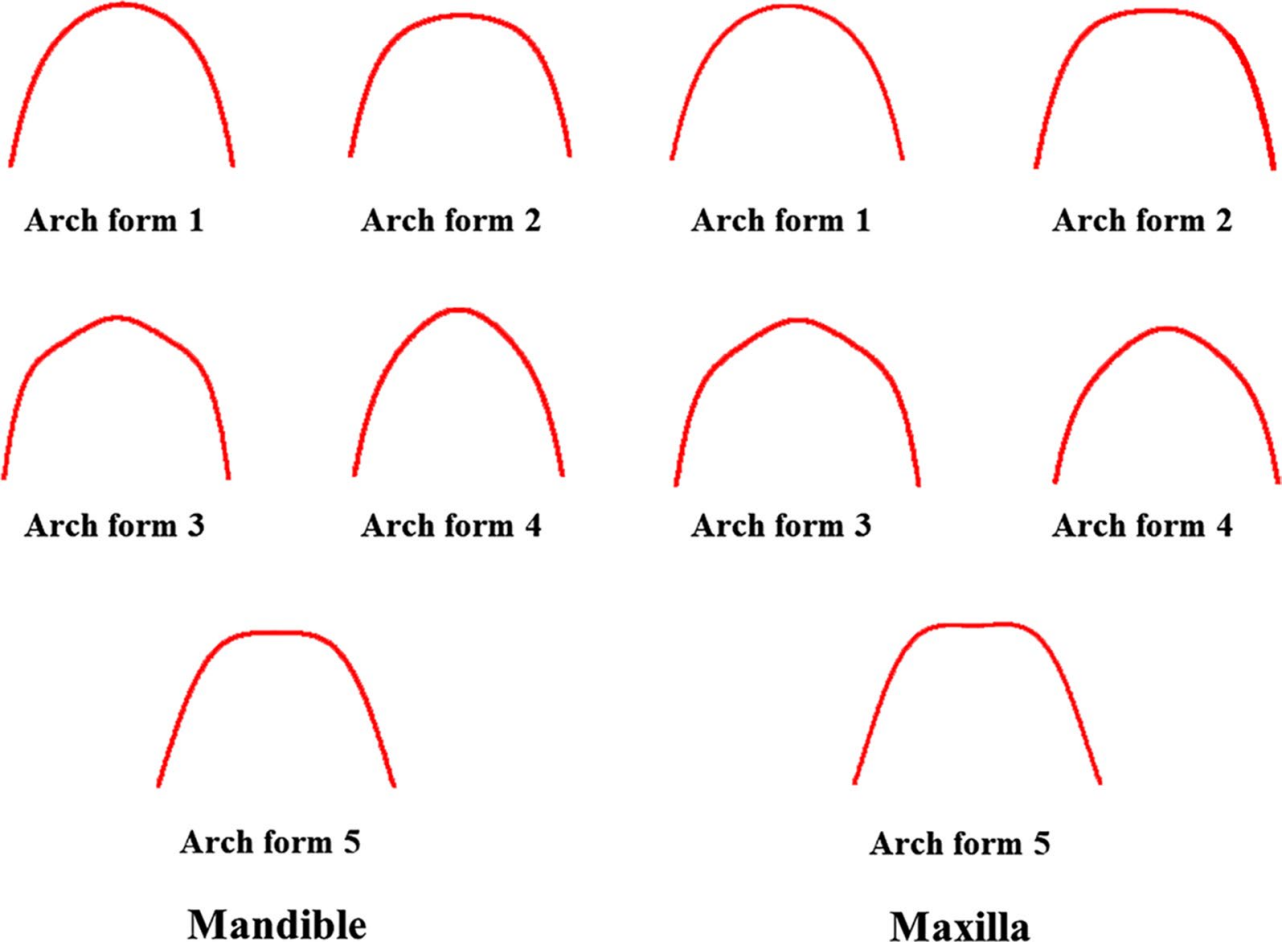
Graphic representations of the 5 dental arch forms for normal occlusion in mandible and maxilla

**Fig. 3:**
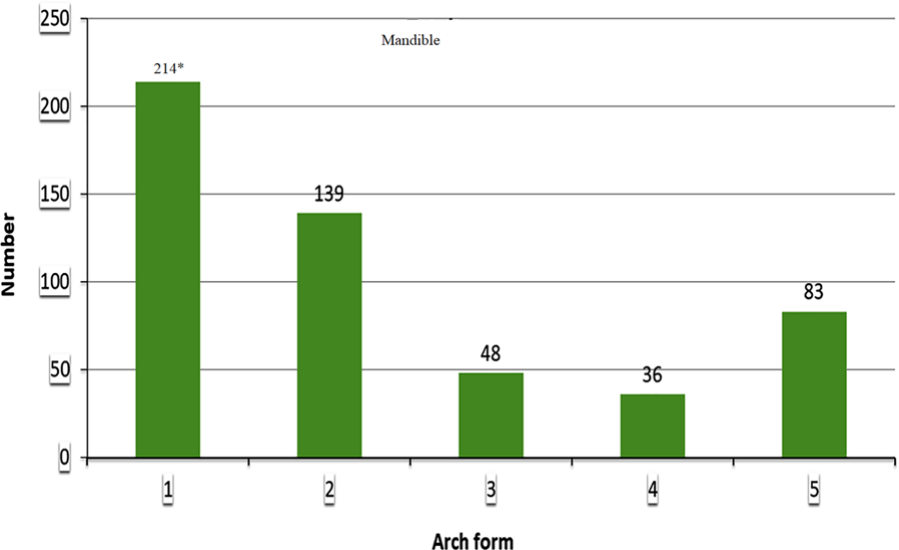
Distribution of subjects in each arch form in the maxilla

**Fig. 4:**
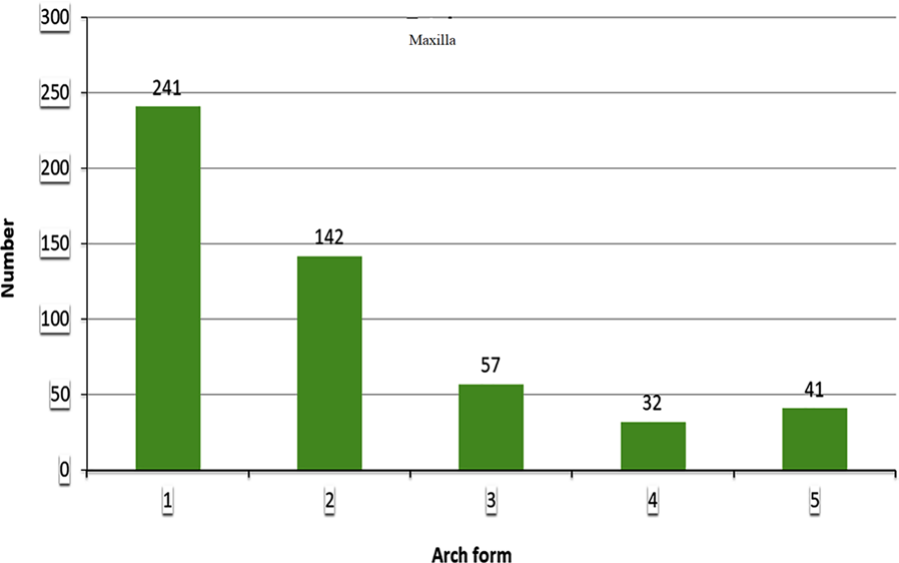
Distribution of subjects in each arch form in the mandible

Five different arch forms were found for each of the maxilla and mandible as shown in Fig. 2. Form 1 was a catenary arch, form 2 was halfway between ellipse and a U-shaped arch, form 3 was a tudor arch, form 4 was a tapered equilateral arch and form 5 was a quadroangular arch. The distribution of subjects across each arch form in the maxilla and mandible are shown in Figs. 3 and 4, respectively. Arch form 1 was the most prevalent form, and was observed in 47% and 41.2% of the maxilla and mandible (Table 2), respectively (*p* ˂ 0.01). Form 4 was the least common in both maxilla and mandible with frequencies of 6.2% and 6.9%, respectively (*p* ˂ 0.01).

**Table 2:** The frequency of each arch form as a percentage in females, males and total sample

	Gender		Total (%)	*P* value
	Female Number (%)	Male Number (%)		
*Mandibular arch form*				
1	127 (43.9)	87 (37.7)	214 (41.2)	0.006*
2	77 (26.6)	62 (26.8)	139 (26.7)	0.358
3	29 (10.0)	19 (8.2)	48 (9.2)	0.149
4	17 (5.9)	19 (8.2)	36 (6.9)	0.739
5	39 (13.9)	44 (19.1)	83 (16.0)	0.583
Total	289	231	520	
*Maxillary arch form*				
1	141 (49.5)	100 (43.9)	241 (47.0)	0.008*
2	63 (22.1)	79 (34.7)	142 (27.7)	0.179
3	39 (13.7)	18 (7.9)	57 (11.1)	0.005*
4	19 (6.7)	13 (5.7)	32 (6.2)	0.289
5	23 (8.1)	18 (7.9)	41 (8.0)	0.435
Total	285	228	513	

Maxillary arch form 1 and 3 were more frequent in females compared to males with *p* ˂ 0.01. For the mandible, arch form 1 was more common in females than males (*p* ˂ 0.01).

### Arch size

**Fig. 5:**
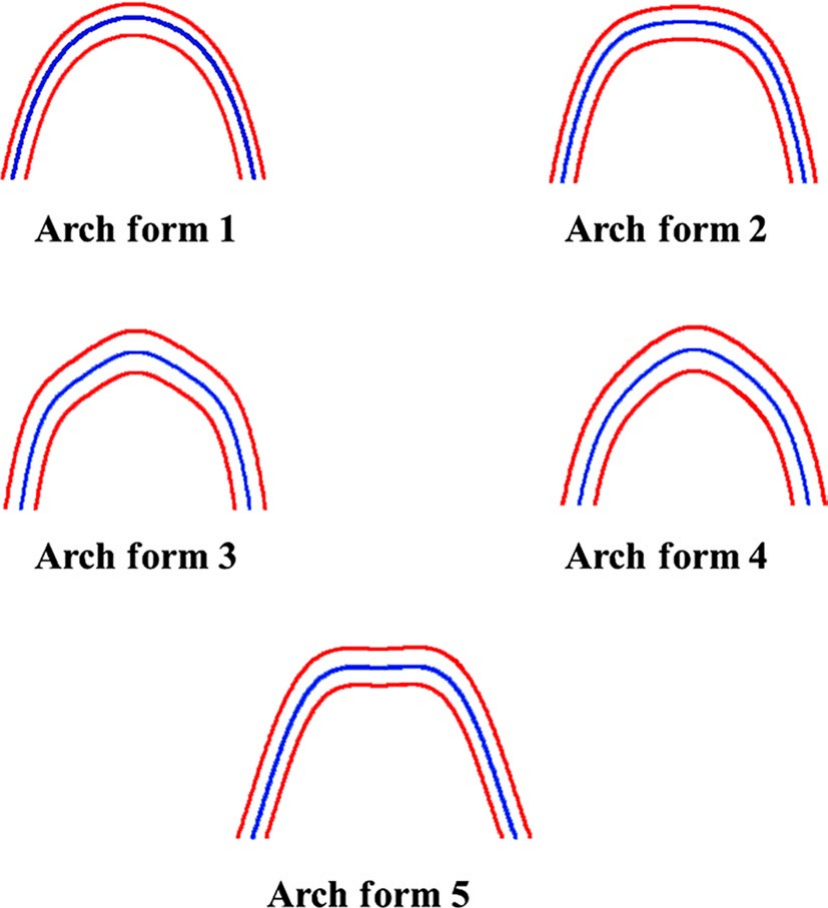
Graphic representations of the 5 arch forms showing 3 sizes: small, medium and large within each arch form

**Fig. 6:**
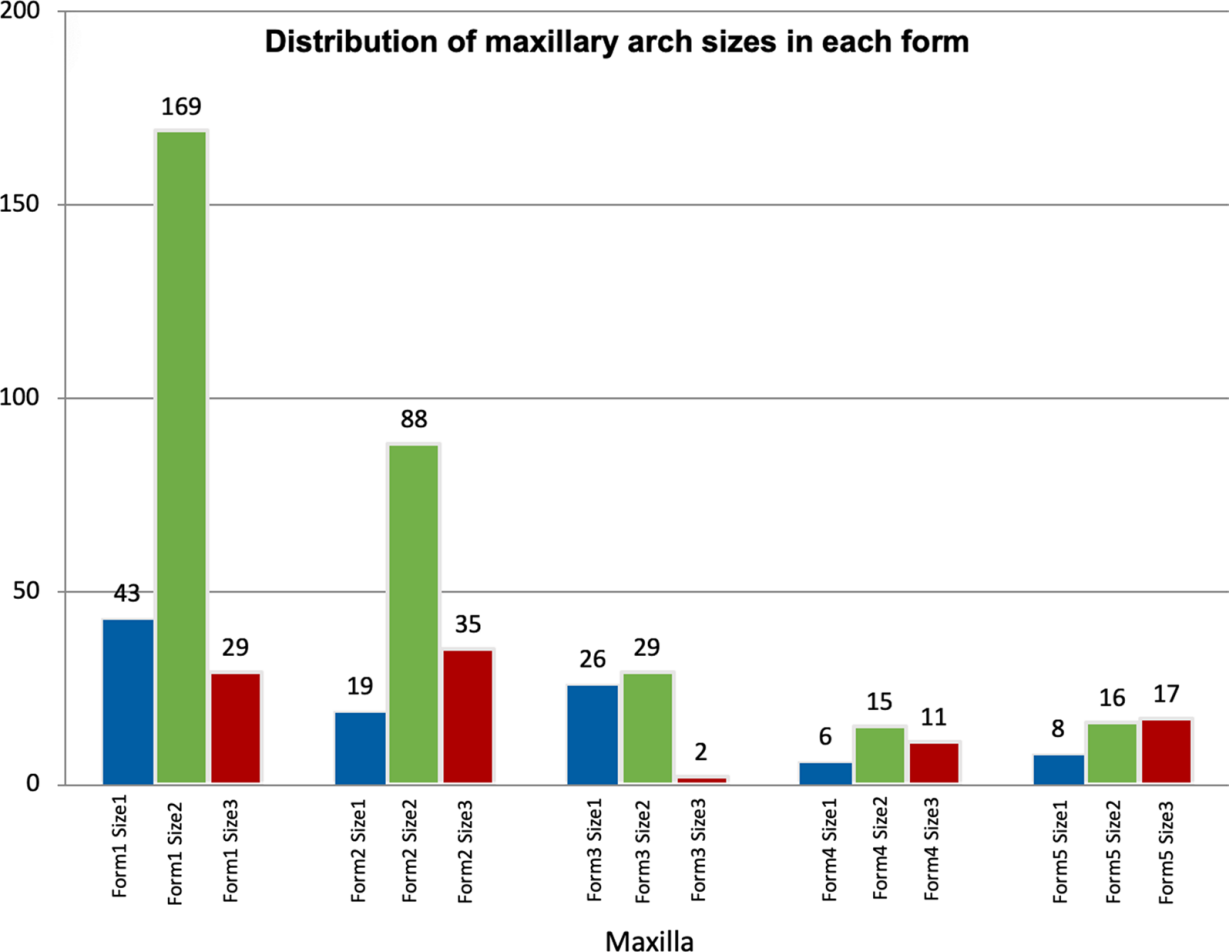
Distribution of maxillary arch sizes in each form

**Fig. 7:**
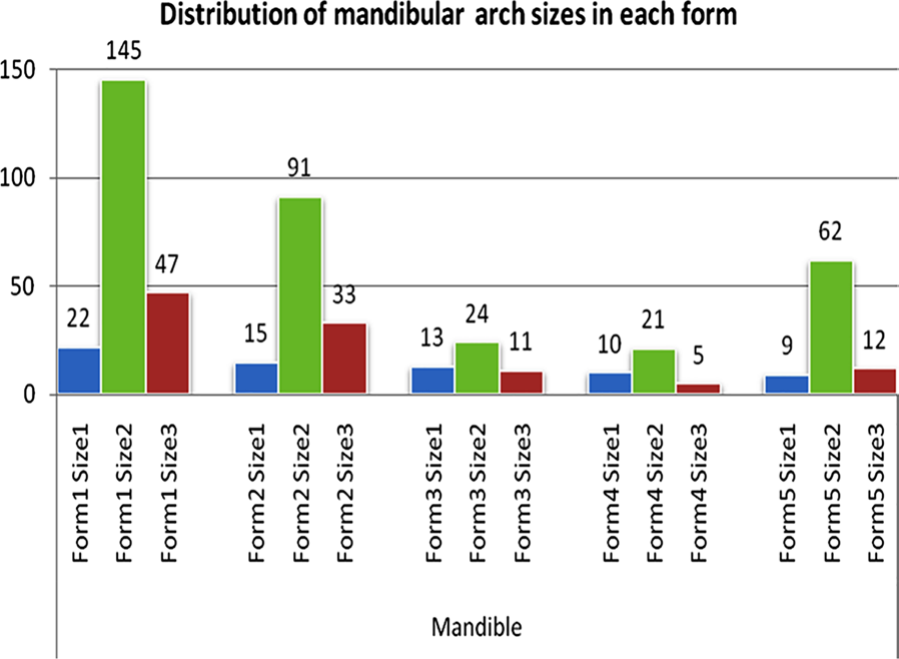
Distribution of mandibular arch sizes in each form

The arch sizes were divided into small, medium and large within each arch form. Figure 5 shows the three arch form sizes. The distribution of subjects in each arch form according to size is shown in Figs. 6 and 7 for the maxilla and mandible, respectively.

The maxillary arch size mean of the total sample was 56.26 ± 2.74 mm for females and 58.32 ± 2.89 mm for males (*p* < 0.001). The mandibular arch size mean was 52.02 ± 2.87 mm and 53.19 ± 3.11 mm for females and males, respectively (*p* < 0.001). Table 3 shows the mean and standard error of the mean (SE) of the 3 comparative arch sizes for males and females.

**Table 3:** Means, standard error of the mean (SE) of the maxillary and mandibular arch sizes in males and females after age adjustment according to ANCOVA test and the significance

Arch	Size	Females Means (SE)	Males Means (SE)	Significance
Maxilla	1	52.89 (0.20)	53.76 (0.31)	0.021
	2	56.99 (0.11)	57.66 (0.13)	0.000
	3	60.58 (0.36)	61.49 (0.21)	0.033
Mandible	1	47.45 (0.25	48.86 (0.36)	0.002
	2	52.24 (0.11)	52.13 (0.13)	0.535
	3	56.03 (0.28)	57.01 (0.23)	0.008

Size 2 (medium) arch was the most prevalent size in males and females, as well as mandible and maxilla (*p* < 0.001).

### Arch parameters

The mean and standard deviation of intercanine and intermolar width and arch depth for each arch form in the mandible and maxilla are presented in Table 4.

**Table 4:** Mean and SD of inter-canine and inter-molar widths (in mm) and arch depth of the maxilla and the mandible

Arch form	Inter-canine width	Inter-molar width	Arch depth
*Mandible*			
1	28.0 ± 2.0	48.1 ± 3.2	30.5 ± 2.6
2	28.9 ± 2.1	48.4 ± 3.2	29.9 ± 2.6
3	28.8 ± 2.0	49.3 ± 3.2	31.4 ± 2.6
4	27.1 ± 2.1	46.6 ± 3.3	29.9 ± 2.7
5	28.8 ± 2.0	47.1 ± 3.2	30.1 ± 2.6
*Maxilla*			
1	35.9 ± 2.3	53.4 ± 2.9	34.5 ± 3.0
2	37.2 ± 2.2	53.6 ± 2.8	34.5 ± 3.0
3	35.1 ± 2.3	52.9 ± 2.9	34.8 ± 3.1
4	34.2 ± 2.4	53.1 ± 2.9	35.1 ± 3.0
5	36.3 ± 2.3	52.0 ± 2.9	33.7 ± 3.0

## Discussion

Identification of a suitable arch form for treating malocclusion is key for achieving a stable, functional, and esthetic occlusion. Clinically, it would be appropriate to have several preformed arch forms that one can choose from for individual patient after identifying the patient’s pretreatment arch form [14].

Although some residual growth might be left between ages 15 and 17, which might be considered by some as a limitation of this study; nonetheless, this age range was used in this study. The main determinants of arch form is intercanine and intermolar width, which exhibit very minimal changes after the age of 9 [20].

The aim of this study was to identify the forms of maxillary and mandibular arches in a Jordanian population. Several studies have been conducted in different populations [10, 13, 21–23]. Most of the studies conducted described the mandibular arch form because the mandible is considered the reference element of diagnosis and treatment in orthodontics [24]. According to several authors, the stability of the form and dimension of the mandibular dental arch is a key factor in predicting the stability of the results [25]. Only few studies focused on maxillary arch [22, 26].

Sixth polynomial function was employed in this study to determine the arch form from the digitized points of tooth positions on the dental arch. It has been reported that the sixth degree polynomial equation is the function that best describe dental arch configuration. The description of some important dental arch regions, such as anterior curvature of the mandibular arch and posterior tooth alignment were adversely affected and compromised by polynomial functions with lower degrees [27].

Five arch forms were identified in each maxilla and mandible in this study. Some previous studies reported three arch forms for their study population (American and Korean population), others reported 5 different arch forms in a French population, while some others reported eight arch forms in a Brazilian population [10, 14, 24].

The high accuracy and objectivity observed in this study was due to the method employed to determine the arch form since grouping of the curves into their corresponding arch forms and sizes was done employing a computer software. Other studies grouped them manually by visual observation or simple calculation [10, 22, 24].

The results of this study showed that there were at least five arch forms that described dental arches among untreated young Jordanian adults with normal occlusion. However, arch form 1 (catenary) was the most prevalent form representing almost half of the samples investigated in this study with a slightly higher frequency in the maxilla compared to the mandible.

Telles [28] reported an elliptical mandibular arch form for the majority of their samples representing almost two thirds of the subjects.

The second most common arch form was form 2, a form halfway between elliptical and U-shaped arch, with a relatively large intercanine distance. About one-quarter of the curves fell under this category. Ricketts [29] reported that one-third of his samples exhibited this arch form while Triviño et al. [10] reported that only 9 percent of the studied mandibular arches belong to this category.

Form 3 was not observed frequently in previous studies. This form has the morphology of the central incisors with a diastema in the posterior region. It was described as "tudor" curve by architects. It was found in about 10% of our samples in both the maxilla and mandible. Triviño et al. [10] reported a higher percentage (18%) of this form in their samples.

Form 4 was observed in a small number of our samples (around 7%). This form has a pointed anterior region. It was described in other studies with different frequencies. Raberin et al. [24] found this form in 19.4% of his French samples while Triviño et al. [10] found it in only 2% of his Brazilian samples.

In form 5, the incisors were arranged in a straight line with initiation of the curvature at the distal region of the lateral incisors, and was described as a quadrangular. This arch form had a low frequency with lower frequency in the maxilla compared to the mandible. Triviño et al. [10] reported a similar figure in the mandible. On the other hand, Triviño and Vilella [30] reported a higher percentage of this arch form and was the predominant form in their study.

The differences in arch forms between our study and other studies could be attributed to the different ethnic backgrounds, sample characteristics and study methodology.

Size 2 (medium) was the predominant size in the maxilla and mandible in almost all the forms. This is a finding that has been reported by other authors [29, 30].

Comparison of the arch forms between females and males showed that there were differences in arch forms 1 and 3 in the maxilla and arch form 1 in the mandible. Other studies reported similar forms but different sizes in both genders [24, 30]. In this study, statistically significant differences in arch size were also found between both genders. These differences, however, were small and could be considered clinically as insignificant.

Differences in the results could be explained by the different ethnic backgrounds and reference points employed for measurements.

The transverse measurements conducted in this study were intercanine and intermolar width. The mean of these parameters were close to the values found in other studies conducted on samples that had semblance with our samples [31]. Other studies reported smaller intercanine width but larger intermolar width [32, 33].

Out of the 5 arch forms that were found in the Jordanian population, two constituted the majority of all arch forms. The rest were less frequent. It is recommended that clinicians keep the most prevalent arch forms as part of their armamentarium. However, if a patient present with one of the less common arch forms in countries with multi-ethnic societies, it is of paramount importance for the clinician to respect the original arch form of this patient. Consequently, for the less common arch forms, clinicians should make adjustments to the archwires according to the patient’s arch form to reduce the likelihood of relapse especially in the intercanine width; since changes in intercanine width is associated with high risk of relapse [25].

It is imperative for further studies to be conducted to ascertain whether the arch forms found in this study can be applied to the whole region considering the similarity in historical background among the surrounding countries.

## Conclusion

Although a generalized arch form for all individuals could not be established, the following conclusions could be drawn:

1. The catenary arch form was the most prevalent form in both the maxilla and mandible; it was more common in females than males.
2. The catenary and the tudor arch forms were more frequent in females than males in the maxillary arch.
3. The medium arch size was the most frequent size in the maxilla and mandible.
4. Males exhibited larger arch size compared to females.

Based on our findings, companies can develop preformed archwires tailored to the needs of the Middle East population.

## Data Availability

All data and materials are available in case the authors are asked to provide them.
