# Correction to: Maxillary and mandibular dental arch forms in a Jordanian population with normal occlusion

**DOI:** 10.1186/s12903-021-01523-1

**Published:** 2021-03-23

**Authors:** M. Aljayousi, S. Al-Khateeb, S. Badran, E. S. Abu Alhaija

**Affiliations:** 1 Division of Orthodontics, Department of Preventive Dentistry, Faculty of Dentistry, Jordan University of Science and Technology, P.O. Box 3030, Irbid, 22110 Jordan; 2 Department of Orthodontics, Faculty of Dentistry, The University of Jordan, Amman, Jordan; 3 College of Dental Medicine, Qatar University, P.O. Box 2713, Doha, Qatar

## Correction to: BMC Oral Health (2021) 21:64 https://doi.org/10.1186/s12903-021-01461-y

After publication of the original article [1], the authors identified an error in Dr. E. S. Abu Alhaija’s affiliations list.

The incorrect affiliations list is:

^1^Division of Orthodontics, Department of Preventive Dentistry, Faculty of Dentistry, Jordan University of Science and Technology, P.O. Box 3030, Irbid 22110, Jordan;

^3^College of Dental Medicine, Qatar University, P.O. Box 2713, Doha, Qatar.

The correct affiliation is:

^3^College of Dental Medicine, Qatar University, P.O.Box 2713, Doha, Qatar.

The original article has been corrected.
